# The chemokine Sdf-1 and its receptor Cxcr4 are required for formation of muscle in zebrafish

**DOI:** 10.1186/1471-213X-7-54

**Published:** 2007-05-22

**Authors:** Shang-Wei Chong, Le-Minh Nguyet, Yun-Jin Jiang, Vladimir Korzh

**Affiliations:** 1 Laboratory of Fish Developmental Biology, Institute of Molecular and Cell Biology, 61 Biopolis Dr., Proteos, 138673, Singapore; 2 Laboratory of Developmental Signaling and Patterning, Institute of Molecular and Cell Biology, 61 Biopolis Dr., Proteos, 138673, Singapore; 3 Department of Biological Sciences, National University of Singapore, 14 Science Dr. 4, 117543, Singapore

## Abstract

**Background:**

During development cell migration takes place prior to differentiation of many cell types. The chemokine receptor Cxcr4 and its ligand Sdf1 are implicated in migration of several cell lineages, including appendicular muscles.

**Results:**

We dissected the role of *sdf1*-*cxcr4 *during skeletal myogenesis. We demonstrated that the receptor *cxcr4a *is expressed in the medial-anterior part of somites, suggesting that chemokine signaling plays a role in this region of the somite. Previous reports emphasized co-operation of Sdf1a and Cxcr4b. We found that during early myogenesis Sdf1a co-operates with the second Cxcr4 of zebrafish – Cxcr4a resulting in the commitment of myoblast to form fast muscle. Disrupting this chemokine signal caused a reduction in *myoD *and *myf5 *expression and fast fiber formation. In addition, we showed that a dimerization partner of MyoD and Myf5, E12, positively regulates transcription of *cxcr4a *and *sdf1a *in contrast to that of Sonic hedgehog, which inhibited these genes through induction of expression of *id2*.

**Conclusion:**

We revealed a regulatory feedback mechanism between *cxcr4a*-*sdf1a *and genes encoding myogenic regulatory factors, which is involved in differentiation of fast myofibers. This demonstrated a role of chemokine signaling during development of skeletal muscles.

## Background

Several cell movements are associated with somitogenesis, including the convergence of lateral mesodermal cells into presomitic mesoderm and later its segmentation. During somite epithelialization two types of cells are formed – epithelial border cells and inner mesenchymal cells. As somite matures, presumptive muscle cells start to express characteristic muscle-specific proteins (MSP) and elongate either actively or through fusion to form myofibrils [1-13]. The border cells undergo migration/rearrangement of their position [14]. The fast muscle cell elongation is triggered by migration of slow muscle cells [5], which in turn is dependent upon Hedgehog (Hh) signaling [15-23]. A high level of Hh induces Engrailed-expressing muscle pioneers, a subset of slow muscle cells located at the horizontal myoseptum, and a small subset of fast fibers, the Engrailed-expressing medial fast fibers. A low level Hh induces superficial slow fibers, which precursors migrate from their initial position adjacent to the notochord laterally through the paraxial mesoderm and become the most superficial muscle fibers [4,24]. Specification of most fast muscle in zebrafish does not show obvious signs of lineage-specific cell migration besides being involved in more general events of convergence, mesenchyme-to-epithelial transition (MET) during somite epithelialization followed later on by cell elongation during formation of myofibrils. The fast myofibrils differentiate specifically from the lateral aspect of somites and this process involves relatively short distance migration of prospective myoblasts. These cells express a subset of genes linked to cell migration and at certain A-P levels are capable of undergoing epithelial-mesenchymal transition (EMT), migrate and establish appendicular muscles [reviewed in [25,26]].

Chemokine receptors are members of the superfamily of seven-transmembrane domain, G-protein coupled receptors. The CXC chemokine receptor CXCR4 [27,28] is used by HIV-1 for binding to the cell membrane [27,29-31]. SDF-1α [chemokine (C-X-C motif) ligand 12; zebrafish gene nomenclature committee] and its receptor CXCR4 [chemokine (C-X-C motif) receptor 4; zebrafish gene nomenclature committee] bind only each other [32-35]. Importantly, a study of the expression of SDF-1α/CXCR4 in the mouse embryo demonstrated expression of CXCR4 in the presomites [36]. The knockout SDF-1α and CXCR4-mice are the only known chemokine/chemokine receptor mutants that display embryonic lethality [37]. They demonstrate defects of cell migration during formation of the neural tube and heart [38,39]. The Sdf1-Cxcr4 interaction also plays a role during the chemotaxis of primordial germ cells in zebrafish in mice [40-42], and sensory cells in zebrafish [43-47].

Our previous study demonstrated that the zebrafish Cxcr4 is encoded by two related genes expressed in a complex pattern, including somites [48]. Later on, it was shown that homologous genes are expressed in human muscle satellite cells and play a role in cell migration during tongue and limb myogenesis in mice [49-51]. While this suggests a role for Cxcr4 in late myogenesis, a role of Cxcr4 in early somitogenesis still remains to be elucidated. Since the zebrafish mutant of *cxcr4b *– *ody *does not show obvious defects in myogenesis [41], we analyzed the second receptor – *cxcr4a*.

Prior to segmentation in zebrafish, myoblasts initiate expression of myogenic regulatory factors (MRFs) [17] important for skeletal muscle commitment and myotube formation [52]. The highly related bHLH proteins MyoD, Myf5, Myogenin and Mrf4 have a pivotal function in muscle cell specification and differentiation [53-57]. They share a common dimerization domain and DNA binding domain (DBD), the basic helix-loop-helix (bHLH) motif. MRFs regulate myogenesis after forming heterodimers with ubiquitous E proteins. These bind to E box, with core consensus sequence of CANNTG, in the promoter of target genes [58]. How these proteins initiate the program of muscle cell differentiation remains to be deciphered explicitly. Recently, MyoD was shown to have repressive activity in presence of other cofactors [59].

In this report, we described the involvement of Cxcr4a and Sdf1a during formation of fast muscles in zebrafish and provided *in vivo *evidence of a role of *cxcr4a*-*sdf1a *in the regulation of MRFs during myogenic determination. The lack of Cxcr4a-Sdf1a-mediated signaling leads to reduction in expression of somitic markers and decrease in fast myofibrils. The lack of Cxcr4a-Sdf1a also affects migration of slow muscle. This effect could be indirect. In addition, we show that E12 and MyoD-Myf5 regulate *cxcr4a*. This suggested a possible feedback loop between *cxcr4a*-*sdf1a *axis and *myoD*/*myf5*. In addition, we discovered that ectopic Hh represses transcription of *cxcr4a *and *sdf1a *through a negative regulator of cell differentiation Id2. Taken together, our data connect MRFs and chemokines in a regulatory relationship during early myogenesis.

## Results

### *cxcr4a *and *sdf1a *are expressed in a dynamic manner during formation of fast muscles

**Figure 1:**
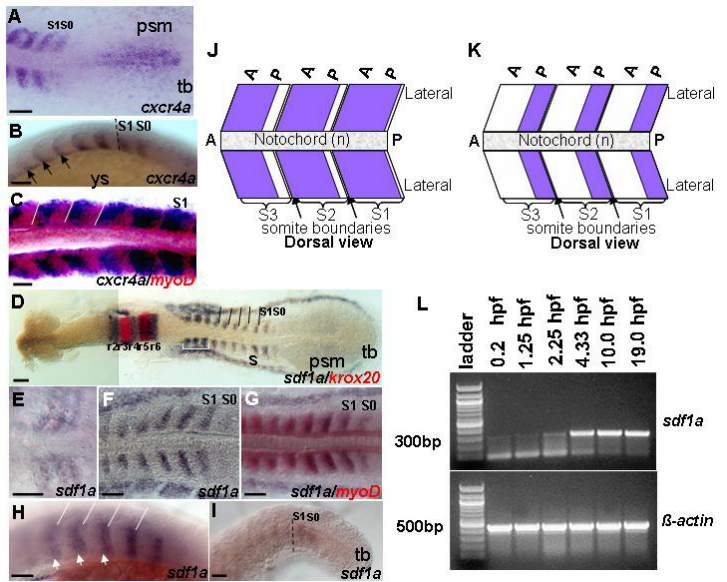
**The dynamic expression of *cxcr4a *and *sdf1a *during segmentation**. Dorsal views (A,C-G,J,K) and lateral views (B,H-I). (A,B) Expression of *cxcr4a *in posterior trunk. (D-I) RNA in situ hybridization with *sdf1a *riboprobe (blue). (A,B) High level of *cxcr4a *transcript in newly formed and posterior somites, 13.5 h and 16 h respectively. In somites, *cxcr4a *expression is restricted to anterior part. Expression becomes increasingly restricted to anterior part within each somite over time, black arrows. (C) Overlapping expression domain of *cxcr4a *with *myoD *(red) is observed, 14 h. (J) Schematic representation of *cxcr4a *expression (blue) in posterior somites. (D) *sdf1a *staining covers almost the entire three anterior-most somites indicated by white bracket, while in posterior somites expression is restricted to posterior part, 13 h. (E) Expression in early somites, 11 h. (F,G) Overlap of expression of *sdf1a *and *myoD *(red), 14 h. (H) Expression is restricted to the posterior part of each somite, 16.5 h, white arrowheads. (I) Faint expression is detected in forming and newly formed somite, 21 h. (K) Schematic representation of *sdf1a *expression (blue) in posterior somites. (L) Reverse transcription (RT)-PCR detects continuous presence of transcript of *sdf1a *during early development. *sdf1a *transcript is present at low levels before mid-blastula transition (MBT). To confirm results, the PCR products were sequenced. *β-actin *was used as a positive control. -RT control using *β-actin *primers without addition of reverse transcriptase, no band was detected (data not shown). Black dashed lines indicate boundary between somite and newly formed somite (B,I). White lines demarcate the somite boundaries (C,H). Abbreviations: a – anterior; p – posterior; psm – presomitic mesoderm; r – rhombomere; s – somite; S0 – forming somite; S1 – newly formed somite; tb – tailbud; ys – yolk sac. Scale bars = 50 μm.

Both *cxcr4a *and *cxcr4b *are expressed during somitogenesis [48]. To better understand the role of Cxcr4 and Sdf1 in the formation of somitic musculature, we re-evaluated the expression pattern of two *sdf1 *and two *cxcr4 *genes. *sdf1a *and *sdf1b *were cloned by PCR. As detected by WISH, transcripts of both *cxcr4a *and *cxcr4b *cover the newly formed somites almost completely, but the level of expression of *cxcr4a *is higher than that of *cxcr4b *(Figures 1A, B; Additional Figures A1A, B (see Additional file 1)) [reviewed in [60]]. The most posterior somite, which is still forming, weakly expresses *cxcr4a*. The next pair of somites that already formed expresses *cxcr4a *at a higher level (Figure 1A). As development proceeds, expression of both *cxcr4a *and *cxcr4b *become restricted to the anterior half of somite (Figure 1B; Additional Figure A1B (see Additional file 1)). It persists until about 22 h when it becomes restricted to the few posterior somites (data not shown). By end of segmentation, *cxcr4a *and *cxcr4b *transcripts are no longer detected by WISH.

Since SDF-1α is the only known ligand of CXCR4 [32,34], we examined the expression pattern of the two zebrafish *sdf1*genes. Both *sdf1a *and *sdf1b *are expressed maternally (Figure 1L; Additional Figure A1H (see Additional file 1)). The level of *sdf1a *transcript increases rapidly from the onset of mid-blastula transition (MBT). In contrast, the level of *sdf1b *transcript increases from fertilization.

During early somitogenesis *sdf1a *is expressed in the lateral somitic mesoderm (Figure 1E). Later, *sdf1a *expression is restricted mainly to the posterior part of somite. However, in the anterior most three somites, *sdf1a *transcripts cover almost the entire somite (Figure 1D). At mid-somitogenesis, only the posterior somites express *sdf1a *(Figure 1H). During late segmentation the forming and newly-formed somites express *sdf1a *at low level (Figure 1I). By 24 h, *sdf1a *expression is no longer detected by WISH (data not shown).

The expression level of *sdf1b *in the somites is low (Additional Figure A1D (see Additional file 1)). It is first observed in the adaxial cells and later become restricted to the posterior part of somite similar to that of *sdf1a*. By mid-somitogenesis, *sdf1b *transcripts are restricted to the dorsal and ventral parts of somites (Additional Figures A1D-G (see Additional file 1)).

To define how *cxcr4 *or *sdf1*are expressed in respect to other markers, we used the two-color WISH. The expression pattern of both *cxcr4a *and *cxcr4b *overlaps almost entirely with that of *myoD *in the forming somite and a few posterior-most somites (*cxcr4a*), but in more mature somites both *cxcr4s *are expressed in the anterior part of somite and *myoD *in the posterior part (Figure 1C; Additional Figure A1C (see Additional file 1)). In contrast to *cxcr4a*, expression pattern of *sdf1a *overlaps completely with that of *myoD *(Figures 1F–G). The expression patterns of *cxcr4a *and *sdf1a *are summarized in a diagram (Figures 1J–K). Therefore, *cxcr4a *and *cxcr4b *are co-expressed with *sdf1a *in the forming and newly formed somites, but not in more mature somites. This suggests that the chemokine and its receptor may have both early and late function during myogenesis.

### *cxcr4a *and *sdf1a *function is required for formation of fast muscles

Based on the fact that *cxcr4 *is expressed during early somitogenesis, we hypothesized that deficiency of Cxcr4 or Sdf1 might affect early myogenesis. To test our hypothesis, we examined somite defects in the mutant *ody*^-/-^, which represents a loss of function of Cxcr4b [41]. There was no obvious somitic defect in *ody*^-/- ^(Additional Figures A2A-D (see Additional file 2)). This could be due to redundancy of *cxcr4b *and *cxcr4a *(Additional Figures A2E-J (see Additional file 2)) [48]. We therefore concentrated our study on Cxcr4a.

**Figure 2:**
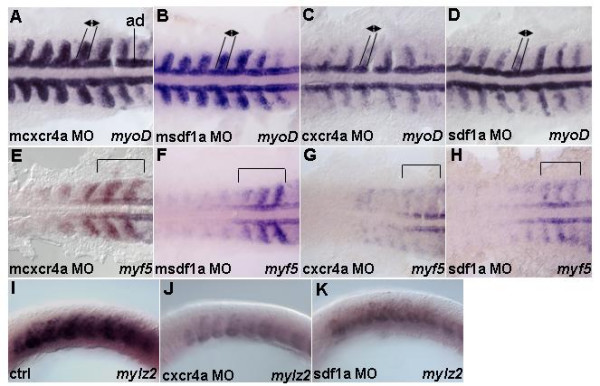
**Cxcr4 signaling is required for transcription of myogenic genes in the paraxial mesoderm**. Dorsal (A-H) and lateral views (I-K). 13 h embryos hybridized with (A-D) *myoD*, (E-H) *myf5 *and (I-K) mylz2 riboprobes. (A,B) m*cxcr4a *(n = 52/52) and m*sdf1a *morphants (n = 50/61) as controls. Embryos show expression pattern of *myoD*. (C) *cxcr4a *(n = 48/50) morphants show *myoD *transcription is reduced in the paraxial mesoderm, while expression in adaxial cells is unchanged. (D) *sdf1a *(n = 50/61) morphants show similar reduction of *myoD *in the paraxial cells but not adaxial cells. Black lines and arrows indicate size of expression domain. In addition, intensity of staining in lateral mesoderm is substantially reduced. (E,F) m*cxcr4a *(n = 36/36) and m*sdf1a *(n = 49/58) morphants as controls. Embryos show characteristic expression pattern of *myf5 *in the adaxial cells, somitic mesoderm and presomitic mesoderm. (G) *cxcr4a *(n = 43/47) morphants have *myf5 *reduced in both somites and forming somites. (H) *sdf1a *(n = 56/71) morphants cause similar effects to Cxcr4a knock down. Black brackets indicate a region where pattern and intensity of *myf5 *staining in the newly formed and forming somites were reduced. (I-K) Control (n = 30). Reduced *mylz2 *transcription in *cxcr4a *(n = 30) and *sdf1a *(n = 30) morphants. Abbreviation: ad – adaxial cells.

Different antisense morpholino oligonucleotides (MOs) designed to target non-overlapping regions of 5'-UTR of both *cxcr4a *and *sdf1a *were injected into one to two-cell stage embryos (morphants). The universal control MO and anti-*cxcr4a*/*sdf1a *MOs with 4–5 base mutations were injected into embryos used as controls. The morphological analysis or acridine orange staining to detect apoptosis or anti-phosphohistone H3 antibody staining to detect cell proliferation did not show obvious changes in somites of morphants (Additional Figure A3 (see Additional file 3) and data not shown). In contrast, *myoD *expression in *cxcr4 *morphants is much reduced in the paraxial cells (Figures 2A, C). Expression of another myogenic bHLH gene, *myf5*, was similarly affected in somites (Figure 2G; Additional Figures A2K-M (see Additional file 2)). Three MOs that targeted 5'UTR of *cxcr4a *caused a similar phenotype (data not shown). Taken together, these results suggested that Cxcr4a plays a role in early myogenesis.

Next we decided to evaluate which of the two ligands, Sdf1a or Sdf1b, co-operates with Cxcr4a during myogenesis. In Sdf1a morphants expression of *myoD *and *myf5 *was down-regulated in somites (Figures 2D, H). In contrast, overall expression of *myoD *and *myf5 *was unaffected in Sdf1b morphants even although in some of them somites were slightly elongated (data not shown). Furthermore, we designed sdf1a-EI-MO which targets the second intron of *sdf1a *causing missplicing of *sdf1a *transcripts as confirmed by electrophoresis and sequencing (Additional Figure A4B (see Additional file 4) and data not shown). The phenotype of sdf1a-EI-MO morphants is similar to that of 5'UTR-*sdf1a *morphants. In addition, expression of genes encoding myosin light chain (*mylz2*) and myosin heavy chain (*myhz1*) decreased in *cxcr4a *and *sdf1a *morphants (Figures 2J–K), whereas expression of the early myocyte marker *pax7 *was relatively normal (data not shown). Taken together, these experiments showed that knockdown of Sdf1a causes reduction in *myoD *and *myf5 *transcription, a phenomenon similar to that of Cxcr4a knockdown. Thus, *sdf1a *is necessary for early myogenesis.

**Figure 3:**
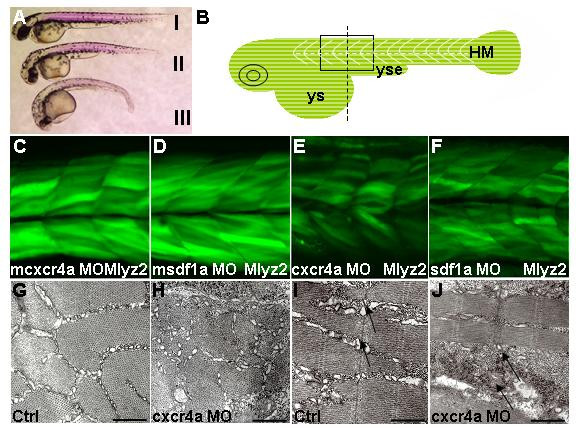
**Formation of fast muscle requires Cxcr4a and Sdf1a**. Lateral views (A-F), cross-section (G,H), sagittal section (I,J) and dorsal views. (A) Birefringence revealed by polarized light in *cxcr4a *(II) and *sdf1a *(III) morphants was reduced compared to control (I), 30 h. (B) Schematic illustrating black box region used for imaging. Start of yolk sac extension as a guide for the center of frame, indicated by dashed line in diagram of zebrafish embryo. (C-F) Single confocal images taken at level of the somite boundary as a guide of depth. Myosin light chain transgenic line, 51 h. (C,D) m*cxcr4a *(n = 87/87) and m*sdf1a *(n = 31/35) morphants developed normally. (E,F) In representative *cxcr4a *(n = 63/71) and *sdf1a *(n = 73/82) morphants, reduction of GFP signal was observed. (G-J) Transmission electron micrograph of cross (G,H) and sagittal (I,J) sections in trunk region of control and *cxcr4a *morphants respectively, 36 h. A representative *cxcr4a *morphant clearly shows a reduction in muscle fibrils. (I,J) Black arrows indicate lack or absence of sarcoplasmic reticulum and muscle fibers in some areas of *cxcr4a *morphant. For clarity, this region of section (J) was selected where there are at least some muscle fibers. Abbreviations: HM – horizontal myoseptum; ys – yolk sac and yse – yolk sac extension. Scale bars = 500 nm.

We then analyzed *cxcr4a *and *sdf1a *morphants in more details. Both *cxcr4a *and *sdf1a *morphants have reduced birefringency in myotomes (Figure 3A). In addition, transgenic *mylz2*-GFP morphants of *cxcr4a *and *sdf1a *show reduced GFP expression (Figures 3B–F). This prompted us to check the ultrastructure of muscle fibers in morphants using transmission electron microscopy (TEM). Both cross and sagittal sections illustrated that myofibrils were reduced in *cxcr4a *morphants (Figures 3G–J). Taken together, these results indicated that deficiency in Sdf1a-Cxcr4a mediated signaling caused abnormal development of skeletal muscles. The affected somitic cells most likely remained undifferentiated.

### Loss of *cxc4a *and *sdf1a *function affects slow muscle migration

**Figure 4:**
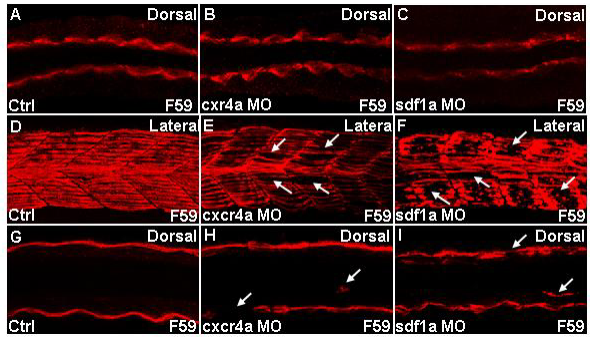
**Slow muscle migration defects in *cxcr4a *and *sdf1a *morphants**. Confocal images of embryos stained for slow myosin using F59 antibody. Dorsal (A-C;G-I) and lateral (D-F) views of embryonic trunk between the fourth and tenth somites. (A-C) Adaxial cells in *cxcr4a *and *sdf1a *morphants are identical to that in controls, 19 h. (D-I) Embryos at 25 h. (D-F) Z-stacked images of ten frames. (G-I) Z-stacked images of two frames. (D) Distinct and properly aligned slow fibers are seen in control embryo. (E,F) Gaps are seen in myotomes of representative *cxcr4a *and *sdf1a *morphant, indicated by white arrows. (G) Control. (H,I) Loss of fiber at the superficial layer and misrouted slow muscle, indicated by white arrows in representative *cxcr4a *and *sdf1a *morphant respectively. Other misrouted slow fibers in morphants are in different planes (data not shown).

It was previously shown that development of slow muscle is closely associated with that of fast muscle and that a change in adhesion within the myotome disrupts migration of slow myoblasts [1]. We tested whether perturbation of either Cxcr4a or Sdf1a affects slow muscle. To eliminate the possibility of early defects in slow myoblasts, we analyzed *cxcr4a *and *sdf1a *morphants at 19 hpf, when the posterior adaxial cells have not yet completed their migration. The adaxial cells in both control embryos and morphants (*cxcr4a *and *sdf1a*) were adjacent to the notochord (Figures 4A–C). A mild decrease in F59 antibody staining in morphants is presumably due to a slight developmental delay. This correlates with the normal *myoD *staining in the adaxial cells (see Figures 2A–D). Normally by 25 hpf slow muscle cells migrate to the lateral edge of somite and align to form myofibrils (Figures 4D, G). In *cxcr4a *and *sdf1a *morphants this process was affected (Figures 4E–F, H–I). Taken together, these results show that while early specification of slow muscle in both *cxcr4a *and *sdf1a *morphants remain normal, the myofibrils were affected.

### *myoD *and *myf5 *are required co-operatively for the expression of *cxcr4a *and *sdf1a*

In mammals, the primary MRFs, Myf5 and MyoD, are involved in both myoblast specification and differentiation [61]. The early expression of *cxcr4a *or *sdf1a *correlates with that of *myoD or myf5*. Therefore, we speculated that the knockdown of MyoD or Myf5 could cause a change in *cxcr4a*/*sdf1a *transcription. Neither injection of the two different *myoD *MO designed against distinct regions at 5'-UTR nor the splice site MO against the first intron of *myoD *(myoD-EI-MO), which effectively inhibits splicing of *myoD *(Additional Figures A4C-F (see Additional file 4)) caused significant changes in transcription of *cxcr4a *and *sdf1a *(data not shown).

**Figure 5:**
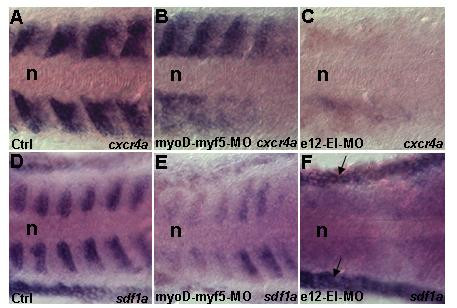
**Knockdown of E-box factors affects *cxcr4a *and *sdf1a *transcription**. Dorsal views (A-F). Embryos between 13–14 h were analyzed. (A,D) Control showing *cxcr4a *(n = 20) and *sdf1a *(n = 20) expression respectively. (B,E) Double *myoD-myf5 *morphants demonstrate reduction of *cxcr4a *(n = 15/20) and *sdf1a *(n = 16/20) transcription respectively. This indicates cooperative function of MyoD and Myf5. (C,F) *e12 *morphants have vast reduction of *cxcr4a *(n = 20/20) and *sdf1a *(n = 20/20) transcription, this confirms that E12 is a major regulating factor. Black arrow in F indicates *sdf1a *staining in the non somitic lateral mesoderm. Abbreviation: n – notochord.

Similarly, the splice site MO against the first intron of *myf5 *(myf5-EI-MO), which caused effective missplicing of *myf5 *(Additional Figures A4G-J (see Additional file 4)), did not affect expression of *cxcr4a *or *sdf1a *(data not shown). This could be due to redundancy of *myf5 *and *myoD *[62]. To explore whether these genes can regulate expression of *cxcr4 *or *sdf1 *cooperatively, we co-injected *myoD *and *myf5 *MOs. Analysis of *myoD-myf5 *double morphants using *cxcr4a *and *sdf1a *probes demonstrated that transcription of these two genes has decreased significantly (compare Figures 5A to 5B and 5D to 5E). The tissue-specific bHLH proteins act after forming dimers with ubiquitously expressed bHLH proteins such as E12. These dimers act as positive regulators of gene transcription. We knocked down E12 using a splice MO (Additional Figures A4K-N (see Additional file 4)). This resulted in strong decrease of *cxcr4a *and *sdf1a *transcription (Figures 5C, F). Other signaling cascades such as Delta-Notch were unaffected by this treatment (Additional Figures A2N-O (see Additional file 2)). Taken together, these results suggest that myogenic factors, MyoD and Myf5 may co-operatively contribute to the regulation of *cxcr4a *or *sdf1a*.

### *myoD *and *myf5 *positively regulate *cxcr4a *transcription

**Figure 6:**
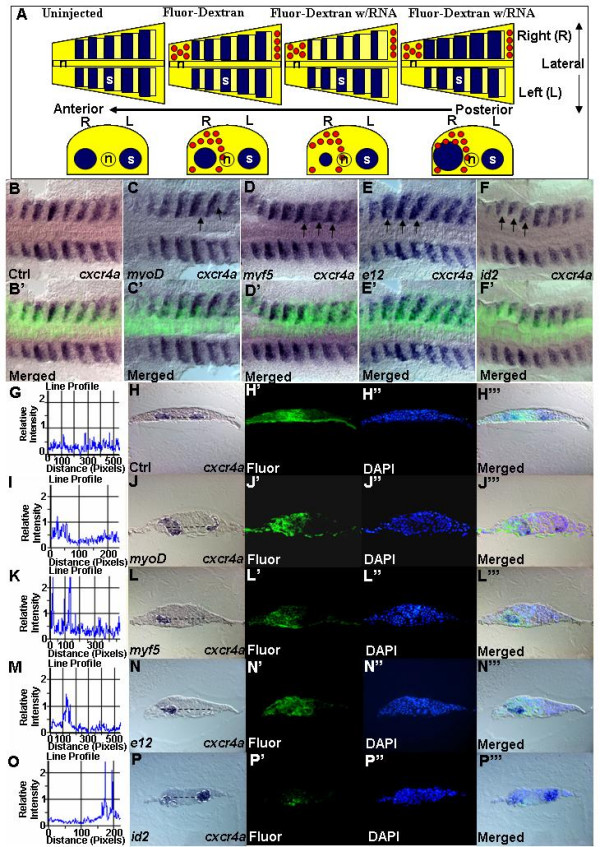
**MRFs and co-factors are required for *cxcr4a *transcription**. (A) Schematic showing the outcome of two-cell stage injection. Blue represents a signal of *cxcr4a *in situ hybridization. Red circles represent fluorescein-dextran on injected side. In control uninjected embryos, transcription analysis with antisense probes will appear symmetrical on left and right sides of flat mount zebrafish embryo. Staining will not differ significantly between left and right sides in both uninjected embryos and control fluorescein-dextran injected embryos. All comparisons were done between opposing pairs of somites. For each set of experiment, a minimum of three embryos between 11–14 h were analyzed using cryosectioning. The uninjected side acts as the internal control. Embryos stained with *cxcr4a *riboprobe. (B-F') Dorsal views. (B'-F') Composite images of the bright-field and fluorescent image showing one sided distribution of mRNA expressing cells. Increased level of *cxcr4a *transcript can be seen after misexpression of *myoD *(n = 46) (C,C'), *myf5 *(n = 35) (D,D') and *e12 *(n = 36) (E,E'). Decreased expression of *cxcr4a* was observed after misexpression of id2  (n = 27) (F,F'). Black arrows indicate sites of effects. Embryos are carefully aligned for cross section. (H,J,L,N,P) Transverse sections at the level of somites. (H',J',L',N',P) Fluorescein-Dextran to indicate location and proper one-sided injection. (H",J",L",N",P") DAPI staining. (H"',J"',L"',N"',P"') Composite images of bright-field and fluorescent images indicate exact site of effect. An increase of *cxcr4a *transcript after misexpression of *myoD *(J), *myf5 *(L), *E12 *(N) and decrease after misexpression of *id2 *(P). White dotted lines demarcate area of staining while black dotted lines define where relative intensities of staining were measured. (G,I,K,M,O) Graphs from Image-Pro Plus software. Control, G. Changes of relative intensity, indicated by peaks in I,K,M and O respectively.

To verify the idea that early MRFs could regulate expression of *cxcr4a*/*sdf1a*, we injected mRNA of *myoD *or *myf5 *into only one cell of the two-cell stage embryo and assayed for *cxcr4a *and *sdf1a *expression during somitogenesis (Figure 6A). To ascertain that mRNA is indeed asymmetrically distributed, the mRNA was co-injected with Fluorescein-Dextran. Only embryos with one-sided distribution of Fluorescein-Dextran were selected for analysis. Ectopic overexpression of *myoD *caused increased transcription of *cxcr4a *(Figures 6C, C'). For detailed analysis these embryos were carefully oriented and sectioned. Analysis of sections confirmed observations made on whole mounts (Figures 6J–J"'). Image-Pro^® ^Plus software was used to evaluate the changes in transcriptional intensities over distance (Figure 6I). Similarly, ectopic overexpression of *myf5 *and *e12 *caused increased transcription of *cxcr4a *(Figures 6D, D', E, E', K, L–L"', M, N–N"').

The negative HLH proteins Id1-4 compete with the positive bHLH factors for dimerization with E12 and E47 by forming inactive dimers. This results in inhibition of transcription of genes – targets of positive MRFs [63,64]. Overexpression of *id2 *[65] in a one-sided fashion resulted in the downregulation of *cxcr4a *or *sdf1a *transcription (Figures 6F, F', O, P–P"'). Taken together, these data suggest that *myoD *or *myf5 *act in parallel with their co-factors to regulate transcription of *cxcr4a *or *sdf1a*.

**Figure 7:**
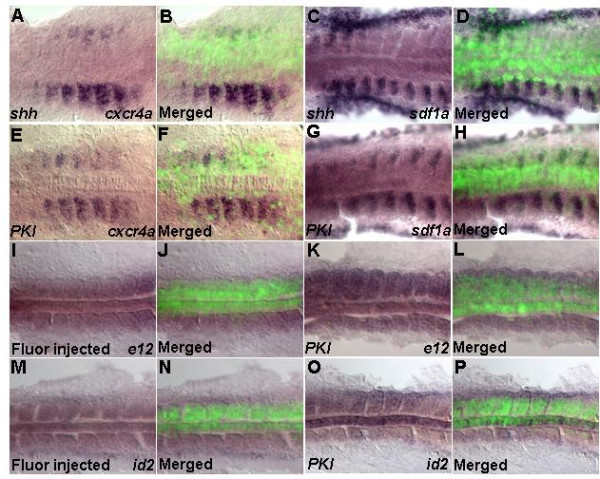
**Shh signaling represses expression of *cxcr4a *and *sdf1a***. Dorsal views (A-P). Ectopic overexpression of *shh *mRNA (100 pg) and *PKI *mRNA (100 pg). Embryos between 11–14 h were analyzed. (A-H) Overexpression of *shh *(A-D) or PKI (E-H) in the somite represses expression of *cxcr4a *and *sdf1a*. (I,J; M,N) Control for *e12 *and *id2 *transcription by fluorescent-dextran injection. (K,L;O,P) Overexpression of *PKI *increases transcription of *e12 *and *id2*.

MyoD, Myf5 and Sdf1 act within a context of Hedgehog (Hh) signaling [22,66-69]. We asked what connection between Cxcr4a-Sdf1a and Shh exists during formation of skeletal muscles. Since transcripts of *cxcr4a *and *sdf1a *were absent from the adaxial cells, we reasoned that this could be due to inhibitory influence of the notochord mediated by Hh. In addition, previously Hh gain-of-function experiments demonstrated transformation of fast myoblasts into slow muscle [6,16,18,20,24]. Thus, we postulated that Hh probably inhibits expression of *cxcr4a *and *sdf1a*. To check this idea, we injected mRNA of *shh *or *PKI*. In agreement with previous reports, this caused an increase in *myoD *and *myf5 *expression (Additional Figures A5A-H (see Additional file 5)) [17,70,71]. Second, ectopic expression of *shh *or *PKI *caused decrease of transcription of *cxcr4a *(Figures 7A–B, E–F) and *sdf1a *(Figures 7C–D, G–H). This could be due to reduction of positive regulators of *cxcr4a*-*sdf1a *transcription or increase of inhibitors. Alternatively, it could also lead to changes in relative levels between both positive and negative regulators. Indeed, overexpression of *PKI *resulted in increased levels of transcription of both positive (*e12*, Figures 7K–L) and negative (*id2*, Figures 7O–P) regulators of *cxcr4a*-*sdf1a*. Taken together, these results suggest that Hh signaling negatively controls expression of *cxcr4a *and *sdf1a*.

## Discussion

We have demonstrated that expression of chemokine receptor Cxcr4a and its ligand Sdf1a in paraxial mesoderm is required during formation of fast muscles. This defines a much earlier role for these molecules in myogenesis comparing to that described in previous reports on the migration of progenitors of the appendicular muscles [25,51,72]. In the gain-of-function (GOF) experiments, based on implantation of SDF1-containing beads into chick limb, the down-regulation of MyoD expression has been observed [51]. Such an outcome differs from that in our observations. This conflict probably demonstrates that an outcome of SDF1 signaling could be the context- and/or concentration-dependent as implied earlier [36]. Furthermore, the different methodological approaches have been used to collect data. We relied on the loss-of-function (LOF) approach, which similar to recent experiments with SDF1 inhibitor [73] demonstrated a positive regulatory role of SDF1-Cxcr4 axis upstream of MyoD. Taken together, results of LOF experiments in chick [73] and our results in zebrafish support the positive regulatory role of Cxcr4-Sdf1 upstream of MRFs during commitment of fast myocytes.

### Functional differences within pairs of Sdf1s and Cxcr4s

Our data show that the both pairs of genes encoding Sdf1 and Cxcr4 have overlapping expression in somites. Neither *cxcr4b *nor *sdf1b *MO alone have obvious effect on formation of fast muscles. There are no defects in this tissue in *ody *mutant. Thus, it appears that Cxcr4a-Sdf1a axis alone is fully capable of supporting fast myogenesis, whereas Cxcr4b or Sdf1b perhaps can only partially compensate for the loss of this activity.

### Cxcr4a-Sdf1a signaling during myogenesis

Both CXCR4 and SDF1 knockout mice exhibit a complex phenotype [74,75]. Despite the fact that they have been available for analysis for a long time, no defects in trunk muscles were detected [50]. Perhaps potential abnormalities in this tissue are too subtle compared to a plethora of more obvious defects elsewhere. Alternatively, an apparent change in regulatory machinery of Cxcr4 and Sdf1 expression between fish and mice, with respect to these proteins in fast muscle development of fish, could explain the differences that we have detected.

We never observed a complete absence of fast fibers in morphants. This could be due to other factors such as an incomplete knockdown or activity of paralogous genes, *cxcr4b *and *sdf1b*, which might partially compensate for the reduction of function of Cxcr4a and Sdf1a. Our data suggest that Cxcr4a-Sdf1a signaling plays no role in tailbud mesoderm and starts to become necessary just before formation of somite border. It has been proposed that Cxcr4b in the lateral line primordium is involved in coordination of internal cell movement [76]. Similarly, Cxcr4a-Sdf1a could be instrumental in coordinating a short distance movement of fast myocytes.

*cxcr4a *and *sdf1a *are not expressed in the adaxial cells and these cells express *myoD *and *myf5 *normally in the *cxcr4a *or *sdf1a *morphants. Thus, the defect of slow myofibrils observed in these morphants is likely an indirect one. It could be caused by abnormality of fast myocytes. This is in line with earlier data suggesting that migration of slow myocytes depends on fast myocytes [5].

### Cxcr4a-Sdf1a and regulation of MRFs expression

The early expression pattern of *sdf1a*-*cxcr4a *in somites is very similar to that of *myoD *and *myf5*, but later on *cxcr4a *expression becomes restricted to the less differentiated anterior part of somite in contrast to MRFs expressed in the posterior more differentiated part of somite. This suggested that developmental events involving Cxcr4 precede induction of expression of *myoD-myf5*. Indeed, our functional analysis illustrated a requirement of Cxcr4 for regulation of expression of MRFs. Thus activation of MRFs expression by *sdf1a*-*cxcr4a *signaling during fast myogenesis occurs concurrently with the process of somite epithelialization and may play a role during this process.

### MRFs positively regulate expression of *cxcr4a *and *sdf1a*

Until now there has been little evidence that MRFs could regulate expression of components of chemokine signaling [76,77]. Here for a first time we provided evidence that MRFs regulate transcription of *cxcr4a *and *sdf1a*. At the same time our results are consistent with previous reports that the MRFs are partially redundant as myogenic determinants [78]. And yet the double MyoD-Myf5 knockdown caused only partial reduction in the expression of *cxcr4a *and *sdf1a*. Perhaps some other regulatory factors such as Myocyte Enhancer Factor 2 (MEF2) act in parallel [54]. Alternatively, these data reflect an incomplete knockdown of MyoD-Myf5.

To act, MRFs form a dimer with E12 and E47, which are products of alternative splicing of *E2A *transcripts. They belong to a distinct class of ubiquitously expressed bHLH proteins of the E-protein family. The MRF/E protein heterodimers bind to a conserved DNA sequence, CANNTG, also known as the E-box, located in regulatory regions of many muscle-specific genes [80-82]. The promoter region of human *CXCR4 *contains an E-box sequence [83]. Similarly, we found two E-boxes in close proximity within a 2 kb stretch of *cxcr4a *5'-untranslated region (our unpublished data). The upstream regulatory sequences of many muscle-specific genes, including MLC1/3 [84], acetylcholine receptor alpha [85], MCK [86] and *myoD *[87], contain multiple E-box sites. In general, at least two E-box sites are required for the activation of these genes by the MRFs [88,89]. These results support an idea that MRFs could directly regulate expression of *cxcr4*.

### Feedback loop between Cxcr4-Sdf1 and MRFs

The expression pattern of Sdf1 genes overlaps with that of MRFs. Since Sdf1a expressed in the posterior part of somite probably interacts with Cxcr4a expressed in the anterior part of somite, this provides a missing link to complete the feedback regulation loop between Cxcr4-MRFs-Sdf1 that could be operating to link cascades of genes involved in chemokine signaling and myogenic differentiation. However, further investigation will be needed to understand the biochemical interactions.

## Conclusion

In summary, our analysis of the developmental role of zebrafish Cxcr4-Sdf1 has led to the identification of the ligand-receptor pair essential for development of trunk muscles. This reveals a novel role of Sdf1-Cxcr4 in differentiation of fast myocytes of the trunk. Thus the chemokine signaling mediated by Sdf1-Cxcr4 emerged as an important regulatory pathway involved in myogenesis.

## Methods

### Zebrafish strains and maintenance

Adult zebrafish (*Danio rerio*) was maintained at 28.5°C as described [10]. The zebrafish AB line (ZIRC) was used as a wild-type fish. The *odysseus *(*ody*^J10049^/cxcr4b) mutants and myosin light chain 2 (*mylz2*-GFP) transgenics were described [6,41]. Pigment formation was blocked with 1-phenyl-2-thiourea (PTU) [65].

### Whole mount in situ hybridization (WISH) and immunohistochemistry

WISH was performed using single-stranded RNA probes labeled with digoxigenin-UTP or fluorescein-UTP (Boehringer Mannheim, Germany) by established protocol. The zebrafish probes *cxcr4a*, *cxcr4b *[48], *myoD *[90], *myf5 *[17,91], *myhz1 *and *mylz2 *[13] have been described previously. Full-length *sdf1a *and *sdf1b *cDNA were obtained by RT-PCR using total RNA and primers designed based on sequence of the EST clones (BM184435 and BM070896), respectively. F59 Mab (1:25) [92] and secondary goat anti-mouse IgG antibody – Alexa Fluor 488 (1:1000; Molecular Probes, USA) was used to detect slow myosin heavy chain.

### RT-PCR

Total RNA was isolated from 14 h embryos using RNeasy^® ^Mini Kit (Qiagen, Germany). cDNA for reverse transcription (RT)-PCR analysis was synthesized using Qiagen^® ^OneStep RT-PCR kit (Qiagen, Germany) and Peltier Thermal Cycler – 200 (MJ Research, USA) according to the manufacturer's instructions. For mRNA splicing analysis, 25 ng of total RNA samples treated with DNAse I was used. RT-PCR conditions were as follows: Reverse transcription 50°C, 30 mins; PCR activation step 95°C, 15 mins; Denaturation 94°C, 1 min; Annealing 59°C, 1 min; Extension 72°C, 1 min; Cycles from Denaturation to Extension were repeated 39 times; Final Extension 72°C, 15 mins. 10 μl of RT-PCR products were loaded into each well for gel-electrophoresis. The sequences of primers for introns of *sdf1a*, *myoD*, *myf5 *and *e12 *were as follows: Forward *sdf1a*-2EI-F 5' ACA GTC AAC ACA GTC CCA CAG 3'; Reverse *sdf1a*-2EI-R 5' GTT GAT GGC GTT CTT CAG GTA 3'; Forward *myoD*-1EI-F 5' CTG AGC AAG GTC AAC GAC GCT 3'; Reverse *myoD*-1EI-R 5' TGA AGT AAG AGC TGT CAT AGC TG 3'; Forward *myf5*-1EI-F 5' GCA CTA CGC CGC TGC ACC T 3'; Reverse *myf5*-1EI-R 5' GCG TCA AAG TTG TAG CTA TTC C 3'; Forward EF1aphaF900 5' CGC CCC TGC CAA TGT AAC CA 3'; Reverse EF1alphaR1388 5' TTG CCA GCA CCA CCG ATT TTC 3'.

### Morpholino (MO) Injections

MOs were obtained from Gene Tools, LLC (USA). The antisense sequences were designed to bind to the 5'UTR region including the initiation methionine or sequence between exon-intron (EI) junctions. To minimize the possibility of non-specific effects, we designed and used at least two MOs targeting non-overlapping sequences for each gene. MO sequences were as follows: Cr4a-1-MO 5' ATA AGC CAT CTC TAA AAG ACT TCT C 3'; Cr4a-2-MO 5' GAC TTC TCC CGT TCC TTC AGT CTC C 3'; Cr4a-3-MO 5' ACA GTT TAA ATA CCT CTC TCG CGC G 3'; mCr4a-1-MO 5' ATA A**A**C CAT **A**TC TAA **G**AG AC**G**TCT C3'; S1a-1-MO 5' TGC AGT GTG AAG AAG AGA TCC GCA C3'; S1a-2-MO 5' TTG AGA TCC ATG TTT GCA GTG TGA A3' [40]; S1a-3-MO 5' ATC ACT TTG AGA TCC ATG TTT GCA 3' [43]; mS1a-2-MO 5' TT**A**AGA T**A**C ATG TTT G**A**A GTG T**A**A A3'; S1a-EI-MO 5' **GTG CAG ATA CTC AC**A TGA CTT GGA A 3'; myoD-1-MO 5' TGC GAT AAC AAG GGG GCG TGA TTT T 3'; myoD-2-MO 5' GTA AGA CAA AGT CCT TCA GAT CCC G 3'; myoD-EI-MO 5' **GTT TCT CAC**CAT GCC ATC AGA GCA G 3'; myf5-EI-MO 5' **GTC ATA TTTAC**C ATG CTC TCT GAG C3'; e12-1EI-MO 5' **GAA AAC ACAC**CG GCC ACA TTA GAA G 3'; e12-3EI-MO 5' **TTC ACA CTC AC**C AGG CCC GGC AGA C 3'; UMO or control MO 5' CCT CTT ACC TCA GTT ACA ATT TAT A 3' [93]. Bold letters either represent base change or region in intron. MOs were diluted using 1× Danieau's solution to 1 mM stock solution or to proper concentration for injection (0.46–1.5 ρmole) and injected into the yolk stream of 1–2 cell stage embryos using a nanoinjecter (WPI, USA). Since several MO give the same results only representative morphants were photographed.

### Expression constructs and RNA

*cxcr4a *(AY057095), *cxcr4b *(AY057094), *sdf1a *(BM184435), *sdf1b *(BM070896), *myoD *(Z36945), *myf5 *(AF270789), and *id2 *(DQ186992) were all cloned into PCRscript (Clontech). Primers used for cloning: Forward *cxcr4a *(F) 5' ATG GCT TAT TAC GAA CAC ATC GT 3'; Reverse *cxcr4a *(R) 5' TTA ACT AGA GTG AAA GCT TGA GAT 3'; Forward *cxcr4b *(F) 5' ATG GAA TTT TAC GAT AGC ATC 3'; Reverse *cxcr4b *(R) 5' CTA ACT CGT CAG TGC ACT GGA 3'; Forward *sdf1a *(F) 5' ATG GAT CTC AAA GTG ATC GT 3'; Reverse *sdf1a *(R) 5' TTA GAC CTG CTG CTG TTG GGC 3'; Forward *sdf1b *(F) 5' ATG GAT AGC AAA GTA GTA GCG C 3'; Reverse *sdf1b *(R) 5' TTA CTC TGA GCG TTT CTT CTT TAT 3'; Forward *myoD *(F) 5' ATG GAG TTG TCG GAT ATC CCC 3'; Reverse *myoD *(R) 5' GCA CTT GAT AAA TGG TTT CC 3'; Forward *myf5 *(F) 5' ATG GAC GTA TTC TCC ACA TC 3'; Reverse *myf5 *(R) 5' TCA CAG TAC GTG GTA AAC TGG T 3'; Forward *id2 *(F) 5' ATG AAG GCA ATA AGC CCA GTG A 3'; Reverse *id2 *(R) 5' TTA ACG GTA AAG TGT CCT GCT G 3'. For microinjection of mRNA, constructs were linearized with Sac II and capped mRNA was synthesized by *in vitro *transcription with T7 RNA polymerase using mMessage mMachine kit (Ambion, USA). Poly-A was added using poly-A polymerase (GE Healthcare, UK). *E12 *expression construct was provided by Dr. J. Campos-Ortega. Zebrafish *sonic hedgehog *(*Shh*) and *PKI *RNA were transcribed from plasmid pPSP64T-zfshh and pPSP64T-PKI provided by Dr. M. Hammerschmidt. The RNA (100 ρg) was co-injected into the yolk of 1–2-cell stage embryos with lysine fixable Fluorescein Dextran or Texas Red (70 kDa, Molecular Probes, USA).

### Cryosectioning and photography

Cryosectioning of embryos was described [65]. Some sections were stained with 1.5 ml of diluted 3.5 μM DAPI (4', 6-diamidine-2-phenylidole-dihydrochloride) for 20 min and washed in PBS 2× 20 min. Axiophot 2 compound microscope or laser scanning confocal microscope LSM 510 (Zeiss, Germany) with software supplied by the manufacturers or AX70 (Olympus, Japan) were used for photography. For image processing Adobe^® ^Photoshop 5.5 and measuring of relative intensities Image-Pro^® ^Plus 4.5.1 software was used.

### Light Microscopy

For analysis of birefringency of the axial skeletal muscle the Olympus Light dissecting microscope equipped with polarizer (Olympus, Japan) was used as described [94]. A plane of polarization was standard in all analysis.

### Electron-microscopy

Embryos were processed using standard protocols [95], embedded in 100% spurr resin and polymerized at 65°C overnight. Ultra-thin sections were cut on a Reichert-Jung ultramicrotome (Germany) and examined under the transmission electron microscope JEM1010 (JEOL, Japan) at 100 kV.

## Authors' contributions

SWC developed the concept, performed all the experiments and wrote the manuscript. LMN analyzed slow muscle. YJJ drafted the manuscript. VK developed the concept, wrote and approved the manuscript. All authors read and approved the manuscript.

## Supplementary Material

Additional file 1 **Additional Figure A1 The dynamic expression of *cxcr4b *and *sdf1b *during segmentation**. Dorsal views (A,C-F) and lateral views (B,G). (A) *cxcr4b *is expressed in the tailbud region, adaxial cells, paraxial mesoderm, 13.5 h. (B) Expression of *cxcr4b *is reduced as differentiation proceed, strong expression is in forming and newly formed somites, 18 h. (C) Two color in situ for *myoD *(red) and *cxcr4b *(blue) reveals partial overlapping expression of *cxcr4b *and *myoD*, 14 h. White lines demarcate the somite boundaries. (E) *sdf1b *transcription starts early in the adaxial cells, 10 h. (D,F) Expression in somites is relatively weak; some part of adaxial and paraxial mesoderm express *sdf1b *at 14 h and 14.5 h respectively. (G) *sdf1b *transcription localizes in dorsal and ventral regions of somites as indicated by black arrowheads, 16.5 h. (H) Reverse transcription (RT)-PCR detects continuous presence of transcript of *sdf1b *during early development. *sdf1b *transcript is present before mid-blastula transition (MBT). To confirm results, products were sequenced. *β-actin *was used as positive control. -RT control using *β-actin *primers without addition of reverse transcriptase, no band was detected (data not shown). Abbreviations: ad – adaxial cells; d – dorsal; lm – lateral mesoderm; mn – motoneurons; ncc – neural crest cells; ps – presomite; psm – presomitic mesoderm; s – somite; S0 – forming somite; S1 – newly formed somite; tb – tailbud; v – ventral; ys – yolk sac. Scale bars = 50 μm. Click here for file

Additional file 2 **Additional Figure A2 Control experiments**. Dorsal views (A-O). (A,B) Embryos double stained with *myoD *(red) and *vasa *(blue), 14 h. *vasa *riboprobe ensures that *ody*^-/- ^was correctly identified since mutant embryos appear phenotypically normal. No significant change in *myoD *was observed in *ody*^-/-^. Arrows indicate the cluster of primodial germ cells (PGCs). (C,D) Embryos double stained with *myf5 *(blue) and *vasa *(red), 14 h. No significant change was detected in the paraxial mesoderm. Insets showing normal cluster of PGCs in *ody *sib and PGCs were found along midline in head region of *ody*^-/-^. (E-J) Two sets of experiment (E-G and H-J) demonstrating redundancy in function between *cxcr4a *and *cxcr4b*. A lower dosage of *cxcr4a *MO was used to obtain normal *myoD *staining but disrupted *myoD *in paraxial mesoderm in *ody *^-/-^. *vasa *(red) helps to identify *ody *^-/-^. White arrowheads in J indicate ectopic expression of *myoD*. (K-M) *cxcr4a *and *sdf1a *morphants show normal transcription of *myf5 *in tailbud domain, 14 h. (N,O) A representative *e12 *morphant stained with *deltaC *as a control for *cxcr4a *and *sdf1a *transcriptional analysis, 14 hpf. Somites are formed in these morphants and notch pathway is unaffected. Click here for file

Additional file 3 **Additional Figure A3 Range of phenotypes in morphants**. (A) Control. (B) *cxcr4a *morphants. (C) *sdf1a *morphants. *cxcr4a *and *sdf1a *MOs act in a dosage dependent manner. Click here for file

Additional file 4 **Additional Figure A4 Efficiency of splice site MOs**. (A,C,G,K) β-*actin *primers used in +RT control. Splice site MOs inhibit splicing in *sdf1a *(B), *myoD *(D), *myf5 *with degradation (H) and *e12 *(L). Total RNA from control (lane1,5), 0.46 pmole/embryo (lane2,6), 0.92 pmole/embryo (lane 3,7), 2.3 pmole/embryo(lane4,8). Splice product sizes are indicated by white asterisks. (E,F) *mylz2 *riboprobe staining on control (n = 20/20) and *myoD-EI *morphant (n = 19/20) respectively [96]. (I,J) *myogenin *riboprobe staining on control (n = 20/20) and *myf5*-*EI *morphant (n = 18/20) respectively [97]. (M,N) *sdf1a *riboprobe staining on *E12*-*EI *morphant (n = 20/20)and morphant rescued with *e12 *mRNA (n = 10/16). Click here for file

Additional file 5 **Additional Figure A5 Control experiments for in vivo effects of PKI on the induction of myogenic transcription**. (A,B) Fluorescent-dextran injected embryos stained with *myoD *(n = 10), 14 h. (C,D) *PKI *injected embryos with robust *myoD *(n = 10), 14 h. (E,F) Fluorescent-dextran injected embryos stained with *myf5 *(n = 10), 14 h. (G,H) *PKI *injected embryos with augmented *myf5 *(n = 10), 14 h. Click here for file

## References

[B1] Cortes F, Daggett D, Bryson-Richardson RJ, Neyt C, Maule J, Gautier P, Hollway GE, Keenan D, Currie PD (2003). Cadherin-mediated differential cell adhesion controls slow muscle cell migration in the developing zebrafish myotome. Dev Cell.

[B2] Denetclaw WF, Christ B, Ordahl CP (1997). Location and growth of epaxial myotome precursor cells. Development.

[B3] Denetclaw WF, Berdougo E, Venters SJ, Ordahl CP (2001). Morphogenetic cell movements in the middle region of the dermomyotome dorsomedial lip associated with patterning and growth of the primary epaxial myotome. Development.

[B4] Devoto SH, Melancon E, Eisen JS, Westerfield M (1996). Identification of separate slow and fast muscle precursor cells in vivo, prior to somite formation. Development.

[B5] Henry CA, Amacher SL (2004). Zebrafish slow muscle cell migration induces a wave of fast muscle morphogenesis. Dev Cell.

[B6] Ju B, Chong SW, He J, Wang X, Xu Y, Wan H, Tong Y, Yan T, Korzh V, Gong Z (2003). Recapitulation of fast skeletal muscle development in zebrafish by transgenic expression of GFP under the *mylz2 *promoter. Dev Dyn.

[B7] Kahane N, Cinnamon Y, Kalcheim C (1998). The cellular mechanism by which the dermomyotome contributes to the second wave of myotome development. Development.

[B8] Kalcheim C, Cinnamon Y, Kahane N (1999). Myotome formation: a multistage process. Cell Tissue Res.

[B9] Kielbowna L (1981). The formation of somites and early myotomal myogenesis in Xenopus laevis, Bombina variegata and Pelobates fuscus. J Embryol Exp Morphol.

[B10] Kimmel CB, Ballard WW, Kimmel SR, Ullmann B, Schilling TF (1995). Stages of embryonic development of the zebrafish. Dev Dyn.

[B11] Neff AW, Malacinski GM, Chung HM (1989). Amphibian (urodele) myotomes display transitory anterior/posterior and medial/lateral differentiation patterns. Dev Biol.

[B12] Venters SJ, Thorsteinsdottir S, Duxson MJ (1999). Early development of the myotome in the mouse. Dev Dyn.

[B13] Xu Y, He J, Wang X, Lim TM, Gong Z (2000). Asynchronous activation of 10 muscle specific protein (MSP) genes during zebrafish somitogenesis. Dev Dyn.

[B14] Henry CA, Hall LA, Hille MB, Solnica-Krezel L, Cooper MS (2000). Somites in zebrafish double mutant for knypek and trilobite form without internal mesenchymal cells or compaction. Curr Biol.

[B15] Barresi MJ, Stickney HL, Devoto SH (2000). The zebrafish slow-muscle-omitted gene product is required for Hedgehog signal transduction and the development of slow muscle identity. Development.

[B16] Blagden CS, Currie PD, Ingham PW, Hughes SM (1997). Notochord induction of zebrafish slow muscle mediated by Sonic hedgehog. Genes Dev.

[B17] Coutelle O, Blagden CS, Hampson R, Halai C, Rigby PW, Hughes M (2001). Hedgehog signalling is required for maintenance of *myf5 *and *myoD *expression and timely terminal differentiation in zebrafish adaxial myogenesis. Dev Biol.

[B18] Currie PD, Ingham PW (1996). Induction of a specific muscle cell type by a hedgehog-like protein in zebrafish. Nature.

[B19] Currie PD, Ingham PW (1998). The generation and interpretation of positional information within the vertebrate myotome. Mech Dev.

[B20] Du SJ, Devoto SH, Westerfield M, Moon RT (1997). Positive and negative regulation of muscle cell identity by members of the hedgehog and TGF-beta gene families. J Cell Biol.

[B21] Hirsinger E, Stellabotte F, Devoto SH, Westerfield M (2004). Hedgehog signaling is required for commitment but not initial induction of slow muscle precursors. Dev Biol.

[B22] Lewis KE, Currie PW, Roy S, Schauerte H, Haffter P, Ingham PW (1999). Control of muscle cell-type specification in the zebrafish embryo by Hedgehog signalling. Dev Biol.

[B23] Roy S, Wolff C, Ingham PW (2001). The u-boot mutation identifies a Hedgehog-regulated myogenic switch for fiber-type diversification in the zebrafish embryo. Genes Dev.

[B24] Wolff C, Roy S, Ingham PW (2003). Multiple muscle cell identities induced by distinct levels and timing of hedgehog activity in the zebrafish embryo. Curr Biol.

[B25] Haines L, Currie PD (2001). Morphogenesis and evolution of vertebrate appendicular muscle. J Anat.

[B26] Neyt C, Jagla K, Thisse C, Thisse B, Haines L, Currie PD (2000). Evolutionary origins of vertebrate appendicular muscle. Nature.

[B27] Feng Y, Broder CC, Kennedy PE, Berger EA (1996). HIV-1 entry cofactor: functional cDNA cloning of a seven-transmembrane, G-protein coupled receptor. Science.

[B28] Loetscher M, Geiser T, O'Reilly T, Zwahlen R, Baggiolini M, Bernhard M (1994). Cloning of a human seven-transmembrane domain receptor, LESTR, that is highly expressed in leukocytes. J Biol Chem.

[B29] Berson JF, Long D, Doranz BJ, Rucker J, Jirik FR, Doms RW (1996). A seven-transmembrane domain receptor involved in fusion and entry of T-cell tropic human immunodeficiency virus type 1 strains. J Virol.

[B30] Mackay CR (1996). Chemokine receptors and T cell chemotaxis. J Exp Med.

[B31] Moore JP, Trkola A, Dragic T (1997). Co-receptors for HIV-1 entry. Curr Opin Immunol.

[B32] Baggiolini M, Dewald B, Moser B (1997). Human chemokines: an update. Ann Rev Immunol.

[B33] Bleul CC, Fuhlbrigge RC, Casasnovas JM, Aiuti A, Springer TA (1996). A highly efficacious lymphocyte chemoattractant, stromal cell-derived factor (SDF-1). J Exp Med.

[B34] Bleul CC, Farzan M, Choe H, Parolin C, Clark-Lewis I, Sodroski J, Springer TA (1996). The lymphocyte chemoattractant SDF-1 is a ligand for LESTR/fusin and blocks HIV-1 entry. Nature.

[B35] Kim CH, Broxmeyer HE (1998). In vitro behavior of hematopoietic progenitor cells under the influence of chemoattractants: stromal cell-derived factor-1, steel factor, and the bone marrow environment. Blood.

[B36] McGrath KE, Koniski AD, Maltby KM, McGann JK, Palis J (1999). Embryonic expression and function of the chemokine SDF-1 and its receptor, CXCR4. Dev Biol.

[B37] Murphy PM, Baggiolini M, Charo IF, Hebert CA, Horuk R, Matsushima K, Miller LH, Oppenheim JJ, Power CA (2000). International union pharmacology. XXII. Nomenclature for chemokine receptors. Pharmacol Rev.

[B38] Ma Q, Jones D, Borghesani PR, Segal RA, Nagasawa T, Kishimoto T, Bronson RT, Springer TA (1998). Impaired B-lymphopoiesis, myelopoiesis and derailed cerebellar neuron migration in CXCR4 – and SDF-1-deficient mice. Proc Natl Acad Sci USA.

[B39] Zou YR, Kottmann AH, Kuroda M, Taniuchi I, Littamn D (1998). Function of the chemokine receptor CXCR4 in haematopoiesis and in cerebellar development. Nature.

[B40] Doitsidou M, Reichman-Fried M, Stebler J, Koprunner M, Dorries J, Meyer D, Esguerra CV, Leung T, Raz E (2002). Guidance of primodial germ cell migration by the chemokine SDF-1. Cell.

[B41] Knaut H, Werz C, Geisler R, Nusslein-Volhard C, Tubingen 2000 Screen Consortium (2003). A zebrafish homologue of the chemokine receptor Cxcr4 is a germ-cell guidance receptor. Nature.

[B42] Molyneaux KA, Zinszner H, Kunwar PS, Schaible K, Stebler J, Sunshine MJ, O'Brien W, Raz E, Littman D, Wylie C, Lehmann R (2003). The chemokine SDF1/CXCL12 and its receptor CXCR4 regulate mouse germ cell migration and survival. Development.

[B43] David NB, Sapede D, Saint-Etienne L, Thisse C, Thisse B, Dambly-Chaudiere C, Rosa FM, Ghysen A (2002). Molecular basis of cell migration in the fish lateral line: role of the chemokine receptor CXCR4 and its ligand, SDF1. Proc Natl Acad Sci USA.

[B44] Gilmour D, Knaut H, Maischein HM, Nusslein-Volhard C (2004). Towing of sensory axons by their migrating target cells in vivo. Neuron.

[B45] Li Q, Shirabe K, Kuwada JY (2004). Chemokine signaling regulates sensory cell migration in zebrafish. Dev Biol.

[B46] Li Q, Shirabe K, Thisse C, Thisse B, Okamoto H, Masai I, Kuwada JY (2005). Chemokine signaling guides axons within the retina in zebrafish. J Neurosci.

[B47] Knaut H, Blader P, Strähle U, Schier AF (2005). Assembly of trigeminal sensory ganglia by chemokine signaling. Neuron.

[B48] Chong SW, Emelyanov A, Gong Z, Korzh V (2001). Expression pattern of two zebrafish genes, *cxcr4a *and *cxcr4b*. Mech Dev.

[B49] Pituch-Noworolska A, Majka M, Janowska-Wieczorek A, Baj-Krzyworzeka M, Urbanowicz B, Malec E, Ratajczak MZ (2003). Circulating CXCR4-positive stem/progenitor cells compete for SDF-1-positive niches in bone marrow, muscle and neural tissues: an alternative hypothesis to stem cell plasticity. Folia Histochem Cytobiol.

[B50] Ödemis V, Lamp E, Pezeshki G, Moepps B, Schilling K, Gierschik P, Littman DR, Engele J (2005). Mice deficient in the chemokine receptor CXCR4 exhibit impaired limb innervation and myogenesis. Mol Cell Neurosci.

[B51] Vasyutina E, Stebler J, Brand-Saberi B, Schulz S, Raz E, Birchmeier C (2005). CXCR4 and Gab1 cooperate to control the development of migrating muscle progenitor cells. Genes Dev.

[B52] Parker MH, Seale P, Rudnicki MA (2003). Looking back to the embryo: defining transcriptional networks in adult myogenesis. Nat Rev Genet.

[B53] Buckingham M, Bajard L, Chang T, Daubas P, Hadchouel J, Meilhac S, Montarras D, Rocancourt D, Relaix F (2003). The formation of skeletal muscle: from somite to limb. J Anat.

[B54] Molkentin JD, Olson EN (1996). Combinatorial control of muscle development by basic helix-loop-helix and MADS-box transcription factors. Proc Natl Acad Sci USA.

[B55] Perry RL, Parker MH, Rudnicki MA (2001). Activated MEK1 binds the nuclear MyoD transcriptional complex to repress transactivation. Mol Cell.

[B56] Pownall ME, Gustafsson MK, Emerson CP (2002). Myogenic regulatory factors and the specification of muscle progenitors in vertebrate embryos. Ann Rev Cell Dev Biol.

[B57] Puri PL, Sartorelli V (2000). Regulation of muscle regulatory factors by DNA-binding, interacting proteins, and post-transcriptional modifications. J Cell Physiol.

[B58] Blackwell TK, Weintraub H (1990). Differences and similarities in DNA-binding preferences of MyoD and E2A protein complexes revealed by binding site selection. Science.

[B59] Mal A, Harter ML (2003). MyoD is functionally linked to the silencing of a muscle-specific regulatory gene prior to skeletal myogenesis. Proc Natl Acad Sci USA.

[B60] Chong SW, Jiang Y-J (2005). Off limits – Integrins holding boundaries in somitogenesis. Trends Cell Biol.

[B61] Rudnicki MA, Jaenisch R (1995). The MyoD family of transcription factors and skeletal myogenesis. Bioessays.

[B62] Rudnicki MA, Schnegelsberg PN, Stead RH, Braun T, Arnold HH, Jaenisch R (1993). MyoD or Myf5 is required for the formation of skeletal muscle. Cell.

[B63] Benezra R, Davis RL, Lockshon D, Turner DL, Weintraub H (1990). The protein Id: a negative regulator of helix-loop-helix DNA binding proteins. Cell.

[B64] Jen Y, Weintraub H, Benezra R (1992). Overexpression of Id protein inhibits the muscle differentiation program: in vivo association of Id with E2A proteins. Genes Dev.

[B65] Chong SW, Nguyen TTH, Chu LT, Jiang YJ, Korzh V (2005). Zebrafish *id2 *developmental expression pattern contains evolutionary conserved and species-specific characteristics. Dev Dyn.

[B66] Borycki A-G, Brunk B, Tajbakhsh S, Buckingham M, Chiang C, Emerson CP (1999). Sonic hedgehog controls epaxial muscle determination through *Myf5 *activation. Development.

[B67] Gustafsson MK, Pan H, Pinney DF, Liu Y, Lewandowski A, Epstein DJ, Emerson CP (2002). Myf5 is a direct target of long-range Shh signaling and Gli regulation for muscle specification. Genes Dev.

[B68] Klein RS, Rubin JB, Gibson HD, DeHaan EN, Alvarez-Hernandez X, Segal RA, Luster AD (2001). SDF-1α induces chemotaxis and enhances Sonic hedgehog-induced proliferation of cerebellar granule cells. Development.

[B69] Schauerte HE, van Eeden FJ, Fricke C, Odenthal J, Strahle U, Haffter P (1998). Sonic hedgehog is not required for the induction of medial floor plate cells in the zebrafish. Development.

[B70] Concordet JP, Lewis KE, Moore JW, Goodrich LV, Johnson RL, Scott MP, Ingham PW (1996). Spatial regulation of a zebrafish patched homologue reflects the roles of sonic hedgehog and protein kinase A in neural tube and somite patterning. Development.

[B71] Hammerschmidt M, Bitgood MJ, McMahon AP (1996). Protein kinase A is a common negative regulator of Hedgehog signaling in the vertebrate embryo. Genes Dev.

[B72] Amthor H, Christ B, Weil M, Patel K (1998). The importance of timing differentiation during limb muscle development. Curr Biol.

[B73] Yusuf F, Rehimi R, Morosan-Puopolo G, Dai F, Zhang X, Brand-Saberi B (2006). Inhibitors of CXCR4 affect the migration and fate of CXCR4-progenitors in the developing limb of chick embryos. Dev Dyn.

[B74] Nagasawa T, Hirota S, Tachibana K, Takakura N, Nishikawa S, Kitamura Y, Yoshida N, Kikutani H, Kishimoto T (1996). Defects of B-cell lymphopoiesis and bone-marrow myelopoiesis in mice lacking the CXC chemokine PBSF/SDF-1. Nature.

[B75] Tachibana K, Hirota S, Lizasa H, Yoshida H, Kawabata K, Kataoka Y, Kitamura Y, Matsushima K, Yoshida N, Nishikawa S, Kishimoto T, Nagasawa T (1998). The chemokine receptor CXCR4 is essential for vascularization of the gastrointestinal tract. Nature.

[B76] Haas P, Gilmour D (2006). Chemokine signaling mediates self-organizing tissue migration in the zebrafish lateral line. Dev Cell.

[B77] Kury P, Greiner-Petter R, Cornely C, Jurgens T, Muller HW (2002). Mammalian achaete scute homolog 2 is expressed in the adult sciatic nerve and regulates the expression of Krox24, Mob-1, CXCR4, and p57kip2 in Schwann cells. J Neurosci.

[B78] Ratajczak MZ, Kucia M, Reca R, Majka M, Janowska-Wieczorek A, Ratajczak J (2004). Stem cell plasticity revisited: CXCR4-positive cells expressing mRNA for early muscle, liver and neural cells 'hide out' in the bone marrow. Leukemia.

[B79] Rudnicki MA, Braun T, Hinuma S, Jaenisch R (1992). Inactivation of MyoD in mice leads to up-regulation of the myogenic HLH gene give myf5 and results in apparently normal muscle development. Cell.

[B80] Murre C, MaCaw PS, Baltimore D (1989). A nes DNA binding and dimerization motif in immunoglobin enhancer binding, daughterless, MyoD, and myc proteins. Cell.

[B81] Davis RL, Cheng PF, Lassar AB, Weintraub H (1990). The MyoD DNA binding domain contains a recognition code for muscle-specific gene activation. Cell.

[B82] Lassar AB, Davis RL, Wright WE, Kadesch T, Murre C, Voronova A, Baltimore D, Weintraub H (1991). Functional activity of myogenic HLH proteins requires hetero-oligomerization with E12/E47-like proteins in vivo. Cell.

[B83] Moriuchi M, Moriuchi H, Margolis DM, Fauci AS (1999). USF/c-Myc enhances, while Yin-Yang 1 suppresses, the promoter activity of *cxcr4*, a co-receptor for HIV-1 entry. J Immunol.

[B84] Wentworth BM, Donoghue M, Engert JC, Berglund E, Rosenthal N (1991). Paired MyoD-binding sites regulate myosin light chain gene expression. Proc Natl Acad Sci USA.

[B85] Piette J, Bessereau JL, Huchet M, Changeux JP (1990). Two adjacent MyoD1-binding sites regulate expression of the acetylcholine receptor alpha-subunit gene. Nature.

[B86] Buskin JN, Hauschka SD (1989). Identification of a myocyte nuclear factor that binds to the muscle-specific enhancer of the mouse muscle creatine kinase gene. Mol Cell Biol.

[B87] Goldhamer DJ, Brunk BP, Faerman A, King A, Shani M, Emerson CP (1995). Embryonic activation of the myoD gene is regulated by a highly conserved distal control element. Development.

[B88] Weintraub H, Davis R, Lockshon D, Lassar A (1990). MyoD binds cooperatively to two sites in a target enhancer sequence: occupancy of two sites is required for activation. Proc Natl Acad Sci USA.

[B89] Bengal E, Flores O, Rangarajan PN, Chen A, Weintraub H, Verma IM (1994). Positive control mutations in the MyoD basic region fail to show cooperative DNA binding and transcriptional activation in vitro. Proc Natl Acad Sci USA.

[B90] Weinberg E, Allende ML, Kelly CS, Abdelhamid A, Murakami T, Andermann P, Doerre OG, Grunwald DJ, Riggleman B (1996). Developmental regulation of zebrafish MyoD in wild-type, no tail and spadetail embryos. Development.

[B91] Chen YH, Lee WC, Liu CF, Tsai HJ (2001). Molecular structure, dynamic expression, and promoter analysis of zebrafish (*Danio rerio*) *myf-5 *gene. Genesis.

[B92] Miller JB, Teal SB, Stockdale FE (1989). Evolutionarily conserved sequences of striated muscle myosin heavy chain isoforms. Epitope mapping by cDNA expression. J Biol Chem.

[B93] Nasevicius A, Ekker SC (2000). Effective targeted gene 'knockdown' in zebrafish. Nat Genet.

[B94] Felsenfeld AL, Walker C, Westerfield M, Kimmel C, Streisinger G (1990). Mutations affecting skeletal muscle myofibril structure in the zebrafish. Development.

[B95] Spurr AR (1969). A low viscosity epoxy resin embedding medium for electron microscopy. J Ultrastruct Res.

[B96] Hammond CL, Hinits Y, Osborn DPS, Minchin JEN, Tettamanti G, Hughes SM (2007). Signals and myogenic regulatory factors restrict *pax3 *and *pax7 *expression to dermomyotome-like tissue in zebrafish. Dev Biol.

[B97] Lee HC, Huang HY, Lin CY, Chen YH, Tsai HJ (2006). Foxd3 mediates zebrafish *myf5 *expression during early somitogenesis. Dev Biol.

