# Supplementary figures and images for: The chemokine Sdf-1 and its receptor Cxcr4 are required for formation of muscle in zebrafish
Source: BMC Dev Biol. 2007 May 22;7:54. doi: 10.1186/1471-213X-7-54 (PMC1904199; doi:10.1186/1471-213X-7-54)

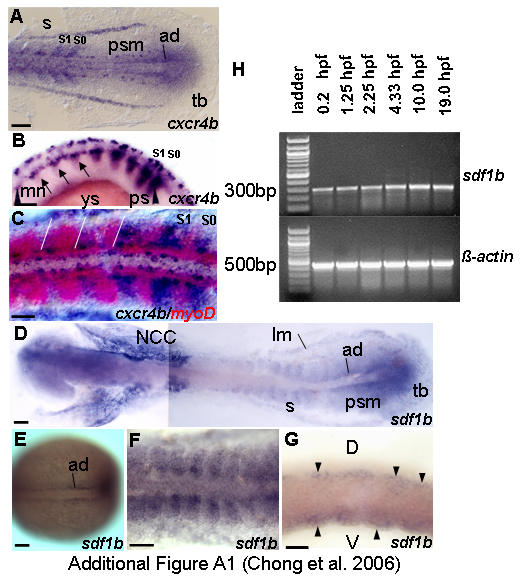

Supplement: Additional file 1 — Additional Figure A1 The dynamic expression of cxcr4b and sdf1b during segmentation. Dorsal views (A,C-F) and lateral views (B,G). (A) cxcr4b is expressed in the tailbud region, adaxial cells, paraxial mesoderm, 13.5 h. (B) Expression of cxcr4b is reduced as differentiation proceed, strong expression is in forming and newly formed somites, 18 h. (C) Two color in situ for myoD (red) and cxcr4b (blue) reveals partial overlapping expression of cxcr4b and myoD, 14 h. White lines demarcate the somite boundaries. (E) sdf1b transcription starts early in the adaxial cells, 10 h. (D,F) Expression in somites is relatively weak; some part of adaxial and paraxial mesoderm express sdf1b at 14 h and 14.5 h respectively. (G) sdf1b transcription localizes in dorsal and ventral regions of somites as indicated by black arrowheads, 16.5 h. (H) Reverse transcription (RT)-PCR detects continuous presence of transcript of sdf1b during early development. sdf1b transcript is present before mid-blastula transition (MBT). To confirm results, products were sequenced. β-actin was used as positive control. -RT control using β-actin primers without addition of reverse transcriptase, no band was detected (data not shown). Abbreviations: ad – adaxial cells; d – dorsal; lm – lateral mesoderm; mn – motoneurons; ncc – neural crest cells; ps – presomite; psm – presomitic mesoderm; s – somite; S0 – forming somite; S1 – newly formed somite; tb – tailbud; v – ventral; ys – yolk sac. Scale bars = 50 μm. [file 1471-213X-7-54-S1.jpeg]

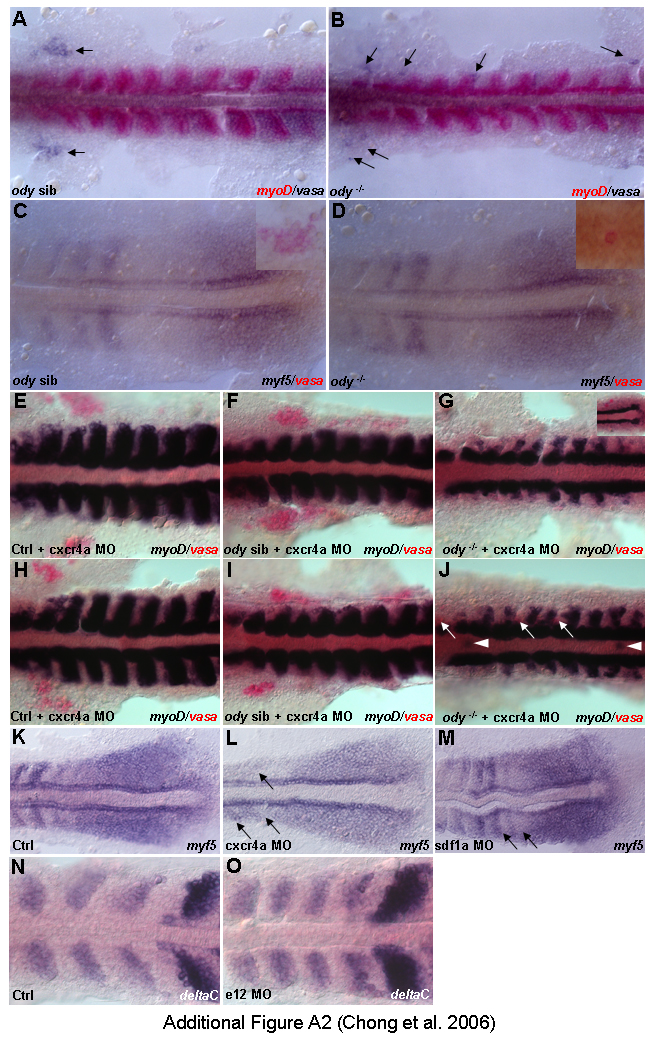

Supplement: Additional file 2 — Additional Figure A2 Control experiments. Dorsal views (A-O). (A,B) Embryos double stained with myoD (red) and vasa (blue), 14 h. vasa riboprobe ensures that ody-/- was correctly identified since mutant embryos appear phenotypically normal. No significant change in myoD was observed in ody-/-. Arrows indicate the cluster of primodial germ cells (PGCs). (C,D) Embryos double stained with myf5 (blue) and vasa (red), 14 h. No significant change was detected in the paraxial mesoderm. Insets showing normal cluster of PGCs in ody sib and PGCs were found along midline in head region of ody-/-. (E-J) Two sets of experiment (E-G and H-J) demonstrating redundancy in function between cxcr4a and cxcr4b. A lower dosage of cxcr4a MO was used to obtain normal myoD staining but disrupted myoD in paraxial mesoderm in ody -/-. vasa (red) helps to identify ody -/-. White arrowheads in J indicate ectopic expression of myoD. (K-M) cxcr4a and sdf1a morphants show normal transcription of myf5 in tailbud domain, 14 h. (N,O) A representative e12 morphant stained with deltaC as a control for cxcr4a and sdf1a transcriptional analysis, 14 hpf. Somites are formed in these morphants and notch pathway is unaffected. [file 1471-213X-7-54-S2.jpeg]

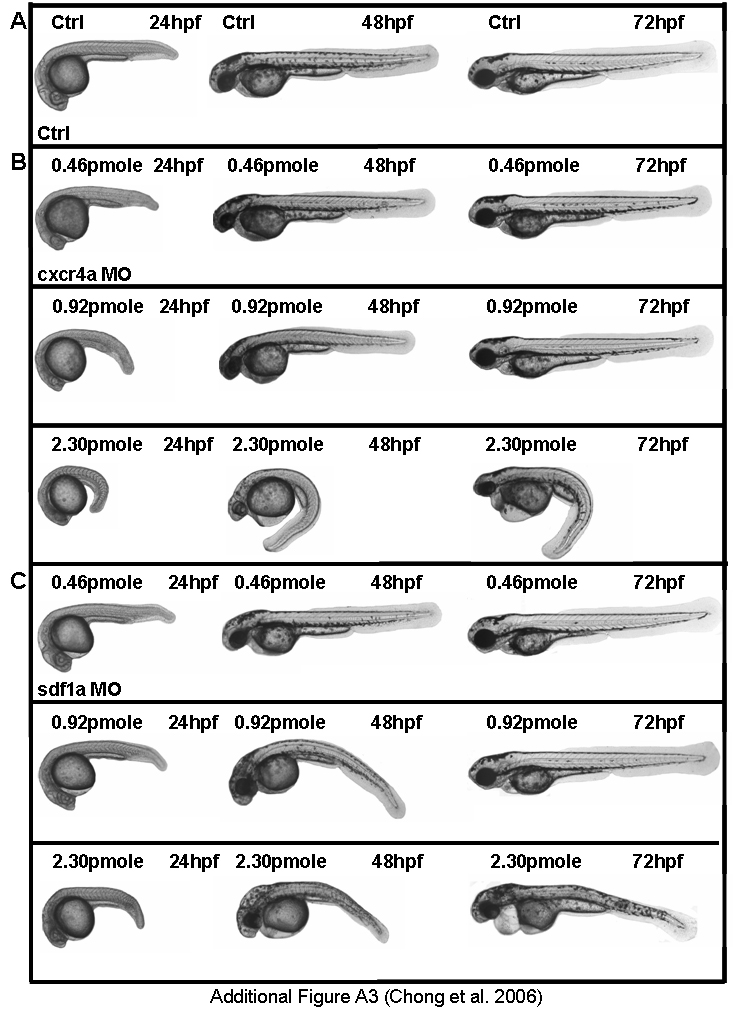

Supplement: Additional file 3 — Additional Figure A3 Range of phenotypes in morphants. (A) Control. (B) cxcr4a morphants. (C) sdf1a morphants. cxcr4a and sdf1a MOs act in a dosage dependent manner. [file 1471-213X-7-54-S3.jpeg]

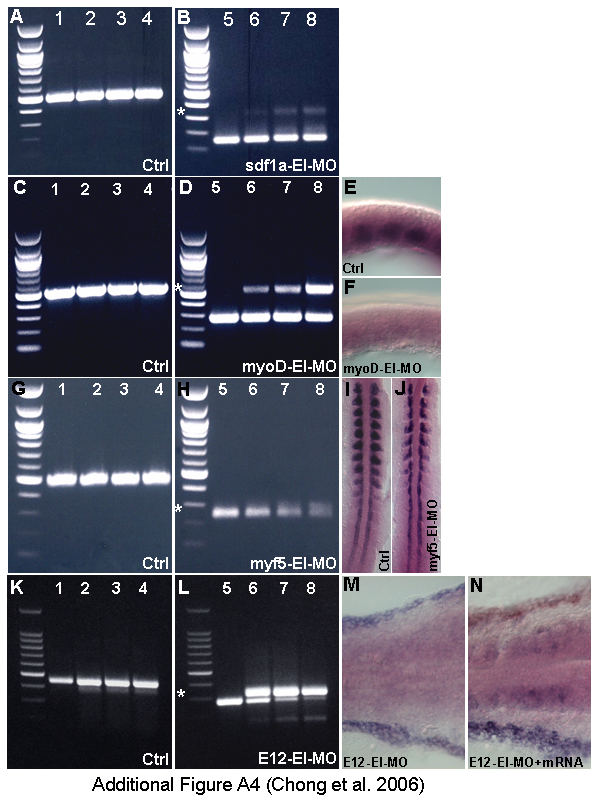

Supplement: Additional file 4 — Additional Figure A4 Efficiency of splice site MOs. (A,C,G,K) β-actin primers used in +RT control. Splice site MOs inhibit splicing in sdf1a (B), myoD (D), myf5 with degradation (H) and e12 (L). Total RNA from control (lane1,5), 0.46 pmole/embryo (lane2,6), 0.92 pmole/embryo (lane 3,7), 2.3 pmole/embryo(lane4,8). Splice product sizes are indicated by white asterisks. (E,F) mylz2 riboprobe staining on control (n = 20/20) and myoD-EI morphant (n = 19/20) respectively [96]. (I,J) myogenin riboprobe staining on control (n = 20/20) and myf5-EI morphant (n = 18/20) respectively [97]. (M,N) sdf1a riboprobe staining on E12-EI morphant (n = 20/20)and morphant rescued with e12 mRNA (n = 10/16). [file 1471-213X-7-54-S4.jpeg]

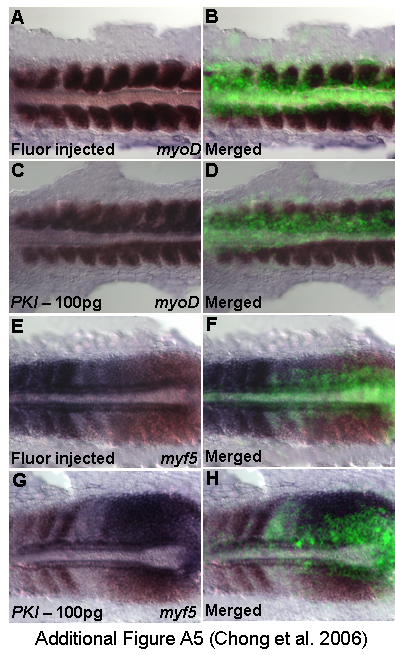

Supplement: Additional file 5 — Additional Figure A5 Control experiments for in vivo effects of PKI on the induction of myogenic transcription. (A,B) Fluorescent-dextran injected embryos stained with myoD (n = 10), 14 h. (C,D) PKI injected embryos with robust myoD (n = 10), 14 h. (E,F) Fluorescent-dextran injected embryos stained with myf5 (n = 10), 14 h. (G,H) PKI injected embryos with augmented myf5 (n = 10), 14 h. [file 1471-213X-7-54-S5.jpeg]
